# Association of Antimicrobial Susceptibility with Treatment Response in *Mycobacterium avium complex* Pulmonary Disease

**DOI:** 10.3390/pathogens14121218

**Published:** 2025-11-29

**Authors:** Xuejiao Luo, Yuhang Chen, Lina Davies Forsman, Yifan He, Guoling Yang, Wei Wei, Hai Lou, Li Wang, Lan Yao, Yidian Liu, Judith Bruchfeld, Jan-Willem Alffenaar, Biao Xu, Xubin Zheng, Wei Sha

**Affiliations:** 1 Clinic and Research Centre of Tuberculosis, Shanghai Key Laboratory of Tuberculosis, Shanghai Pulmonary Hospital, School of Medicine, Tongji University, Shanghai 200433, China; 2 School of Exercise and Health, Shanghai University of Sport, Shanghai 200438, China; 3 Division of Infectious Diseases, Department of Medicine, Karolinska Institute, 17177 Stockholm, Sweden; 4 Department of Infectious Diseases, Karolinska University Hospital, 17176 Stockholm, Sweden; 5 Faculty of Medicine and Health, School of Pharmacy, The University of Sydney, Sydney, NSW 2006, Australia; 6 Westmead Hospital, Syndney, NSW 2145, Australia; 7 The University of Sydney Infectious Diseases Institute (Sydney ID), Sydney, NSW 2145, Australia; 8 Department of Epidemiology, School of Public Health and Key Laboratory of Public Health Safety, Fudan University, Shanghai 200030, China

**Keywords:** *Mycobacterium avium complex*, pulmonary disease, minimum inhibitory concentration, treatment outcome

## Abstract

Understanding the clinical implications of minimum inhibitory concentration (MIC) may facilitate optimal drug selection in *Mycobacterium avium complex* pulmonary disease (MAC-PD) treatment. This study aimed to investigate the association of individual MICs with treatment response of MAC-PD. A retrospective cohort study was conducted in China, including eligible patients diagnosed with MAC-PD between 2018 and 2021. Treatment success rates were calculated across different MIC levels in a subgroup of patients receiving relatively uniform regimens. Associations between MICs and treatment outcomes were investigated by logistic regression analysis. In total, 209 patients with confirmed MAC-PD and initiated treatment were included. The median age was 60.0 years. Among 155 patients who completed treatment, 67.1% achieved treatment success. The treatment success rate was low in patients with clarithromycin MIC ≥ 64 mg/L (25.0%, 1/4) or ethambutol MIC > 16 mg/L (42.9%, 3/7), while remaining relatively stable (75–100%) at other MIC levels. Univariate analyses showed that clarithromycin and ethambutol MICs above these thresholds were associated with increased risk of treatment failure. Our findings suggest an association between clarithromycin MICs and treatment outcomes in patients with MAC-PD receiving standard guideline-recommended regimens. Meanwhile, elevated ethambutol MICs exhibited potential clinical relevance, warranting further investigation.

## 1. Introduction

Nontuberculous mycobacteria (NTM) infection and disease are increasing worldwide [1,2], particularly amongst vulnerable populations, such as those with underlying pulmonary diseases, immunodeficiency, and old age [3,4]. Clinically relevant infection with *Mycobacterium avium complex* (MAC) is very common in China, accounting for 61% of total NTM pulmonary diseases [4]. Current treatment for MAC pulmonary disease (MAC-PD) is mainly based on a three-drug regimen composed of a macrolide, a rifamycin, and ethambutol [5,6,7]. However, this standard regimen seems to be inefficient considering the long treatment duration of at least 12 months after mycobacterium culture conversion, with a low treatment success rate of 61% and a high recurrence rate of 30% [8,9,10].

Appropriate selection of antimycobacterial drugs for MAC-PD treatment based on minimum inhibitory concentrations (MIC), as suggested by the guidelines from major international infectious disease societies and the Clinical and Laboratory Standards Institute (CLSI) [5,7,11], is challenging due to limited understanding of the role of in vitro antimicrobial susceptibility testing [12]. Clinically relevant MIC breakpoints are currently only available for the macrolides based on limited clinical data [13,14,15], while their use remains controversial for amikacin [16,17]. Another two drugs with available MIC breakpoints in MAC-PD treatment are moxifloxacin and linezolid, but their clinical correlations are not established [18,19]. Rifamycin and ethambutol are considered as companion drugs for prevention of acquired macrolides resistance, and no MIC breakpoints have been determined [11]. Due to inherent technical variation in MIC testing and the lack of evidence linking clinical outcomes to specific MIC values within the wild-type distribution of a species [20,21,22], the European Committee on Antimicrobial Susceptibility Testing recommends that MICs should not be treated as absolute values, particularly when they fall within what is considered the susceptible range. This is supported by a recent study from South Korea, which found that treatment responses were comparable when clarithromycin MICs were within the susceptible range but tended to worsen when MICs exceeded it [23].

Given the scarcity of drugs in the pipeline for MAC-PD, it is crucial to find ways to more effectively combine and utilize the existing drugs. The efforts to correlate MIC with treatment outcome are of value to facilitate optimal drug selection and antimicrobial stewardship. Thus, we conducted this retrospective cohort study in one of the largest tuberculosis designated hospitals in China, to investigate the association of individual MICs with treatment response of MAC-PD.

## 2. Materials and Methods

### 2.1. Study Design and Participants

**Figure 1:**
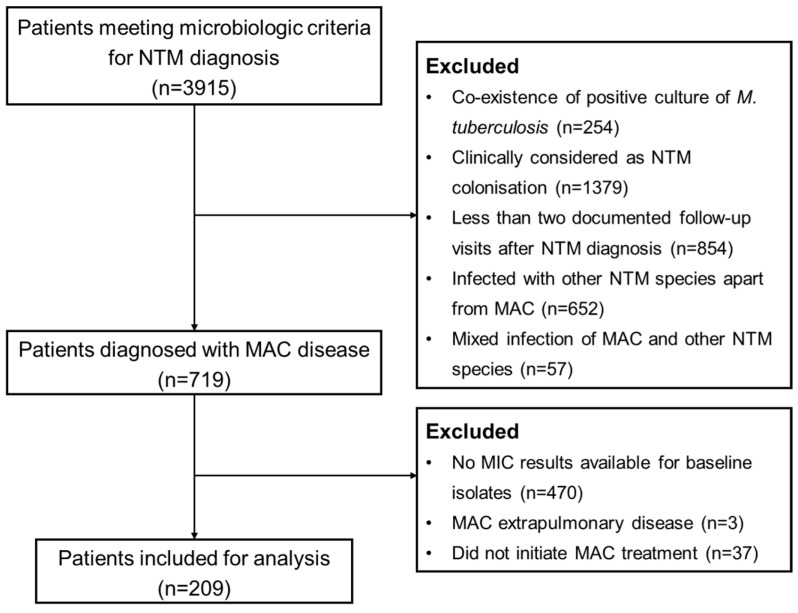
Flowchart for enrolment of patients with *Mycobacterium avium complex* (MAC) pulmonary disease. NTM: nontuberculous mycobacteria; MIC: minimum inhibitory concentration.

A retrospective cohort study was conducted at Shanghai Pulmonary Hospital, which had 196 specialized beds for mycobacterial diseases, to include patients diagnosed with MAC-PD from January 2018 to June 2021. Follow-up data were collected up to June 2024. Records of patients meeting microbiologic criteria for NTM diagnosis were screened, i.e., having at least two positive culture results from separate expectorated sputum samples collected on the same day or on different days, or at least one positive culture results from bronchial wash or lavage [5,6]. Patients were excluded if they had mixed infections with *Mycobacterium tuberculosis* or other NTM species, were clinically considered to have NTM colonization, had extrapulmonary MAC disease, lacked MIC data for the baseline isolate, did not initiate MAC treatment, or had fewer than two documented follow-up visits after NTM diagnosis (Figure 1).

### 2.2. MAC Treatment and Management

The design of the drug regimen was based on clinical practice guidelines from major international infectious disease societies and Chinese national guidelines for NTM-PD treatment [5,6]. In brief, the standard regimen consisted of a macrolide, a rifamycin, and ethambutol. For patients with advanced/severe bronchiectatic or macrolide-resistant MAC-PD, parenteral amikacin was added for at least 2–3 months. Treatment regimens were adjusted at the start or during therapy due to concerns about intolerance and toxicities of antimycobacterial drugs at the discretion of the treating clinician [5,6]. Fluoroquinolones were used as a substitution in MAC-PD treatment regimen when facing aforementioned concerns.

Patients who received a regimen containing at least two antimycobacterial drugs were considered to have started MAC-PD treatment and were followed up once a month to monitor adverse drug reactions and clinical progress. As required by the clinical guidelines, treatment should last for at least 12 months after culture conversion [5,6]. Mycobacterium culture is crucial to monitor treatment response [5,6]; however, respiratory samples were not always collected due to lack of expectoration and bronchial lavages. As an alternative, chest radiology is recommended by the Chinese national guidelines to evaluate clinical improvement [6], with a frequency of once every three months until treatment completion. To minimize radiologic exposure, low-dose computerized tomography scans were performed. Medical records were reviewed to collect clinical information.

### 2.3. Species Identification and Drug Susceptibility Testing

A BACTEC MGIT 960 system (Becton, Dickinson, Franklin Lakes, NJ, USA) was applied for mycobacterium culture. Species identification was performed using a MeltPro Mycobacteria Identification Kit (Zeesan Biotech, Xiamen, China), a licensed commercial kit in China [24]. A high-throughput broth microdilution plate (Sensititre SLOMYCO Susceptibility Testing Plate, Trek Diagnostic System, Thermo Fisher Scientific, Waltham, MA, USA) was used for antimicrobial susceptibility testing from early 2018 [25]. Reference isolates *Mycobacterium peregrinum ATCC 700686* or *Staphylococcus aureus ATCC 29213* were included in each test run for quality control. MIC results reading was performed automatically with the Sensititre Vizion (Thermo Fisher Scientific, Waltham, MA, USA) after 7–14 days, depending on the growth [25]. Results were considered valid and reported only when the MIC values of the reference isolates fell within the reference ranges [25].

### 2.4. Definitions of Treatment Outcome and Main Variables

MAC-PD treatment regimen used for analysis in this study was defined as the main regimen consistently administrated for at least half of the treatment course, with the addition of amikacin as per the modification already specified and administrated for at least two months. The definition of treatment outcome was based on a consensus statement from NTM-NET [26], but modified to fit the national guidelines [6] and current clinical practices in China (Table 1). On the basis of consecutive negative mycobacterial cultures from respiratory samples collected at least 4 weeks apart, culture conversion and microbiological cure were defined. Clinical cure was defined based on resolution of radiologic findings rather than symptoms. Patients who met both microbiological and clinical cure were defined as cured. Patients with progressive pulmonary lesions were classified as treatment failure, even if microbiological samples were unavailable. Those under regular follow-up during treatment but without radiologic or microbiological tests available in study hospital were defined as unevaluable. Treatment success was defined as cure, microbiological cure, or clinical cure, while unfavorable outcome included treatment failure, lost to follow-up, or treatment halted. The classification of radiographic presentations was according to the guidelines from the American Thoracic Society [27]. Briefly, the fibrocavitary form was characterized by cavitary opacities and pleural thickening, predominantly involving the upper lobes, whereas the nodular bronchiectatic form was defined by multifocal bronchiectasis accompanied by clusters of small nodules.

### 2.5. Statistical Analysis

Drugs in the same antibiotic classes were combined to simplify the analyses, apart from rifabutin as the Sensititre plate included rifabutin MICs. Characteristics were compared between groups using the Chi-square test, Fisher’s exact test, or the Wilcoxon rank-sum test, as appropriate, at a two-sided significance level of 0.05. The diversity of drug regimens was visualized using the *UpSetR* package (version 1.4.0). To reduce the potential influence of regimen heterogeneity, a subgroup analysis was performed, restricted to patients who received the standard regimen (a macrolide, rifamycin, and ethambutol), with or without fluoroquinolone and/or amikacin, and completed the full treatment course. Patients who were lost to follow-up, discontinued treatment, or had unevaluable outcomes were excluded from this analysis. Differences in MAC subspecies, radiographic presentation, baseline inflammatory markers, and drug MICs between treatment success and failure were illustrated using the *ComplexHeatmap* package (version 2.22.0). Treatment success rates were calculated across different MIC levels in patients who received the corresponding drugs. Associations between MICs and treatment outcomes were assessed using univariate logistic regression models for exploratory purposes, including the breakpoints suggested by the CLSI guidelines [11]. Multivariable analyses were not conducted due to the limited sample size in the subgroup. All analyses were performed with R software, version 4.1.1 (R Foundation for Statistical Computing).

## 3. Results

### 3.1. Study Patients

Of the 246 patients with confirmed MAC-PD and baseline MIC results, 209 initiated MAC treatment at the discretion of the treating clinician (Figure 1). The median age was 60.0 (IQR: 50.0–66.0) years and 30.1% of them were male. Comorbidities were reported in 74 patients (35.4%). *M. intracellulare* was the dominant subspecies with a proportion of 79.9%. Radiologic data were available for 182 patients, showing that the majority had bronchiectasis (88.5%). Specific to radiographic presentation, noncavitary nodular bronchiectatic disease was the most common form (53.8%, 98/182), followed by cavitary nodular bronchiectatic (25.3%, 46/182), and fibrocavitary (10.4%, 19/182) (Table 2).

### 3.2. Treatment Regimen

**Figure 2:**
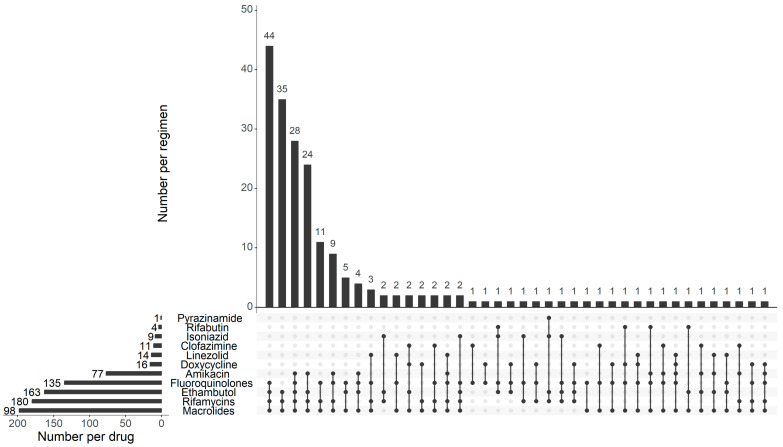
Distribution of drugs and drug regimens in patients initiating treatment for *Mycobacterium avium complex* pulmonary disease (n = 209). The bars represent the number of patients being treated with that drug or drug regimen.

The composition of the treatment regimen was presented for patients initiating MAC treatment (Figure 2). Macrolides were prescribed to most of the patients (n = 198, 94.7%), followed by rifamycin (n = 180, 86.1%), ethambutol (n = 163, 78.0%), fluoroquinolone (n = 135, 64.6%), and amikacin (n = 77, 36.8%). The median number of drugs composing a treatment regimen was 4 (IQR: 3–4) and 98.6% of patients received a regimen containing ≥3 drugs. A standard regimen of a macrolide, a rifamycin, and ethambutol was given to 35 (16.7%) patients, while an additional fluoroquinolone, amikacin, or both were administered to 44 (21.1%), 11 (5.3%), and 28 (13.4%) patients, respectively.

### 3.3. Distribution of Minimum Inhibitory Concentrations

The modal MIC values for clarithromycin, rifampicin, ethambutol, amikacin, moxifloxacin, linezolid, rifabutin, isoniazid, ciprofloxacin, ethionamide, doxycycline, streptomycin, and sulfamethoxazole/trimethoprim were 1, >8, 8, 8 (16), 4, 32, 0.5, >8, >16, >20, >16, 64, and 4/76 mg/L, respectively. According to breakpoints suggested by CLSI guidelines [11], 94.7% of MAC isolates were defined as susceptible to clarithromycin, 80.4% were defined as susceptible to amikacin, while only 9.6% were defined as susceptible to moxifloxacin and 5.8% susceptible to linezolid (Figure S1).

### 3.4. Treatment Outcome and Risk Factors

During treatment courses, 105 patients (50.2%) had more than two follow-up culture results, while 57 patients (27.3%) had no follow-up culture results available. Overall, treatment success was seen in 49.8% of patients (104/209), consisting of 51 cure (24.4%), 12 microbiological cure (5.8%), and 41 clinical cure (19.6%), while treatment failure was seen for 51 patients (24.4%). A total of 23 patients were lost to follow-up during MAC treatment and another 21 patients permanently halted the treatment due to toxicity or personal reasons like poor adherence. 10 patients did not have any microbiological or radiologic outcome documented, and thus were classified as unevaluable (Table 3). The median treatment duration for patients initiating MAC treatment was 15.7 (IQR: 11.9 to 19.3) months, with median durations of 17.1, 17.8, 6.2, 7.3, and 19.2 months for the treatment success, failure, lost to follow-up, treatment halted, and unevaluable groups, respectively.

Overall, 155 patients completed the full treatment course, of whom 104 (67.1%) achieved treatment success. Among the 98 patients with sufficient microbiologic data for evaluation, 63 (64.3%) achieved cure or microbiologic cure. In total, 92 patients had sufficient microbiologic data for evaluation and available radiologic results. Of the 60 patients who demonstrated radiologic improvement, 51 (85.0%) achieved microbiologic cure. Conversely, among the 32 patients without radiologic improvement, 23 (71.9%) had persistent positive cultures.

As shown in Table S1, patients who were lost to follow-up, halted treatment, or had unevaluable outcomes were older, had higher baseline erythrocyte sedimentation rates, and had a higher proportion of prior NTM treatment history compared with those who completed the full treatment course (all *p* values < 0.05). Compared to patients with successful MAC treatment, those with treatment failure or unfavorable outcomes were older, had a higher proportion of comorbidities, pulmonary cavitations and cavitary nodular bronchiectatic form, more extensive pulmonary lesions, and fewer prescribed antimycobacterial drugs (all *p* values < 0.05) (Table S2).

### 3.5. Effect of Drug MICs on Treatment Outcomes

**Figure 3:**
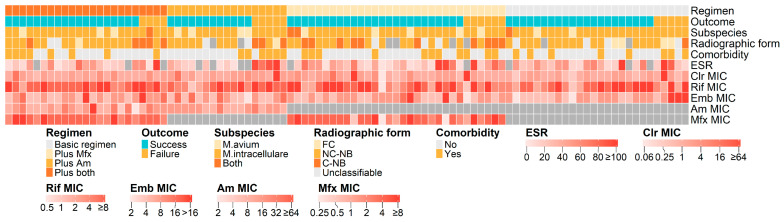
Heatmap of clinical features, bacterial subspecies, minimum inhibitory concentrations (MICs), and treatment outcomes in a subgroup of patients with *Mycobacterium avium complex* pulmonary disease (n = 97). The subgroup included patients who received the standard regimen (a macrolide, rifamycin, and ethambutol), with or without fluoroquinolone and/or amikacin, and completed the full treatment course. Each column represents an individual patient, and dark gray indicates missing values. Clr, clarithromycin; Rif, rifampicin; Emb, ethambutol; Mfx, moxifloxacin; Am, amikacin; FC, fibrocavitary; NC-NB, noncavitary nodular bronchiectatic; C-NB, cavitary nodular bronchiectatic; ESR, erythrocyte sedimentation rate.

We performed a subgroup analysis of patients who received the standard regimen, with or without fluoroquinolone and/or amikacin, and were defined as treatment success or failure (n = 97). As shown in Figure 3, patients who failed treatment were more likely to present with the cavitary nodular bronchiectatic form on radiology and demonstrated higher inflammation at baseline, as reflected by an elevated erythrocyte sedimentation rate. In contrast, no clear difference in MAC subspecies distribution was observed between patients with treatment success and those with treatment failure.

**Figure 4:**
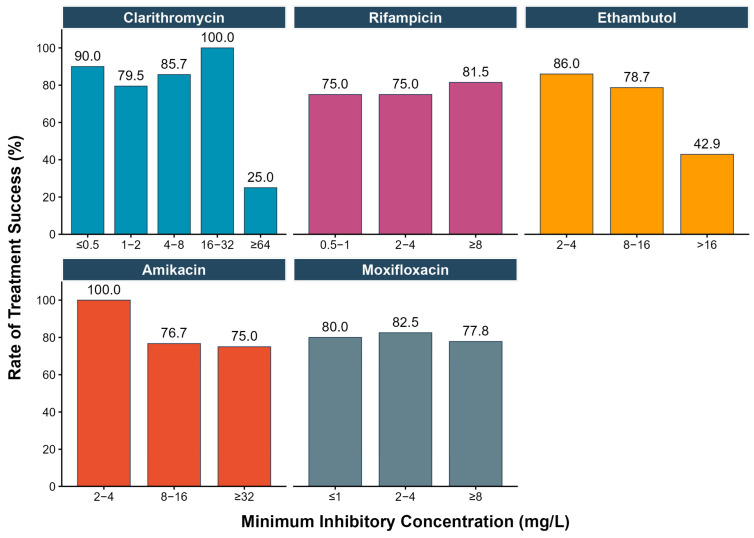
Treatment success rates across different minimum inhibitory concentration (MIC) levels of key antimycobacterial drugs in a subgroup of patients with *Mycobacterium avium complex* pulmonary disease (n = 97). The subgroup analysis was limited to patients who received the standard regimen (a macrolide, rifamycin, and ethambutol), with or without fluoroquinolone and/or amikacin, and completed the full treatment course.

As shown in Figure 4, the treatment success rate was low among patients with strains having clarithromycin MIC ≥ 64 mg/L or ethambutol MIC > 16 mg/L, although the number of study participants was limited. This effect was more pronounced when accounting for regimen diversity: among the seven patients with ethambutol MIC > 16 mg/L, all three who received the standard regimen failed treatment (100%), whereas only one of four patients receiving standard regimen with fluoroquinolone and/or amikacin experienced failure (25%). Consistently, univariate analyses showed that clarithromycin (OR = 13.4, 95%CI 1.61–280.4) and ethambutol (OR = 6.17, 95%CI 1.25–34.0) MICs above these thresholds were associated with increased risk of treatment failure. Such associations were not observed when applying the CLSI-recommended breakpoints (Table 4).

## 4. Discussion

In this retrospective cohort study, we evaluated the association between MICs of key antimycobacterial drugs and treatment outcomes in patients with MAC-PD. The rate of treatment success was found to be low when clarithromycin MICs were ≥64 mg/L in patients receiving standard guideline-recommended regimens, despite the limited sample size. Amikacin and ethambutol MICs also showed potential clinical relevance, while outcomes remained relatively consistent across different MIC levels for moxifloxacin and rifampicin.

A decline in treatment success was observed when clarithromycin MIC reached ≥64 mg/L, a value much higher than the CLSI-recommended breakpoint of 8 mg/L [11]. Due to limited clinical data linking MICs to outcome, current breakpoints are defined mainly on the basis of epidemiological cut-off values (ECOFFs), which represents the highest MIC value for the phenotypic wild-type distribution in vitro [20]. However, susceptibility defined by in vitro MIC values does not always correlate with improved clinical outcomes. In clinical practice, treatment for MAC-PD requires concurrent administration of multiple antimycobacterial drugs, resulting in complex synergistic and antagonistic effects and drug–drug interactions [18,28,29]. For example, concurrent administration of rifampicin and a macrolide has a bidirectional interaction, leading to increased exposure of rifampicin and decreased exposure of macrolide [18,30]. Clarithromycin concentrations may decrease by up to 68% in vivo, with mean peak concentration declining from 3.91 to 1.25 mg/L under concomitant rifampicin therapy [18]. Assuming that clarithromycin achieves approximately 30-fold-higher concentrations in lung tissue relative to plasma [18], the estimated peak concentration in the lung would be around 37.5 mg/L. Although a simplified estimate, this suggests that, when co-administered with rifampicin, clarithromycin is unlikely to achieve effective concentrations against isolates with MICs ≥ 64 mg/L, which also helps explain why a recent South Korean study observed poorer outcomes in patients infected with strains exhibiting clarithromycin MICs ≥ 32 mg/L [23]. Since rifapentine is generally better tolerated than rifampicin, most patients in this study (71.1%, 69/97) received 450 or 600 mg of rifapentine twice weekly, depending on body weight, which may have had less impact on clarithromycin exposure. It may help explain the preserved treatment efficacy observed in patients whose strains had clarithromycin MICs between 16 and 32 mg/L.

Another decline of treatment success rate was observed in patients with strains having ethambutol MICs above 16 mg/L. Similar findings were reported by a previous study from South Korea that the rate of treatment success started to fall when strains having ethambutol and rifampicin MICs ≥ 8 mg/L [31]. Schildkraut et al. demonstrated the value of ethambutol as a backbone drug in a two-drug regimen [32]. It is generally believed that ethambutol is a companion drug in the MAC treatment regimen, which plays a key role in preventing the emergence of resistance by inhibiting cell wall synthesis [7,33], while its role in treatment efficacy is poorly understood. Further studies are needed to validate the role of ethambutol MICs in MAC-PD treatment, and to explore the mechanisms behind it. For amikacin, the treatment success rate tends to be stable across different MIC levels but slightly higher when it falls within 2 to 4 mg/L. The clinical value of amikacin MICs has been reported by several studies and remains controversial [16,17]. We did not observe a significant drop in treatment success rate when amikacin MICs were above the CLSI-recommended breakpoint of 16 mg/L [11]. This might be explained by the differences in administration route, since the breakpoint from CLSI is specified for intravenous infusion of amikacin, a route that requires a high level of patient adherence when administered for at least two months or even longer. In clinical practice, nebulized amikacin is often used as an alternative, which might lead to a higher concentration at infection sites, but the equivalence to intravenous infusion needs further investigation [34].

Associations of rifamycin and fluoroquinolone MICs with treatment outcomes were not significant when assessed individually, highlighting the limited predictive value of their MICs for treatment outcome in MAC-PD. The role of rifampicin in MAC-PD treatment remains controversial. As the study from South Korea showed, patients with strains having ethambutol and rifampicin MICs ≥ 8 mg/L had increased risk of treatment failure [31]. Despite rifampicin having synergistic activity with ethambutol in vitro, it is thought unlikely to be associated with clinical efficacy at current dosing [19,29]. A more recent study challenges the role of rifampicin, with evidence from a hollow-fiber model that rifampicin did not add to the antimycobacterial effect of the two-drug therapy or suppress the emergence of resistance [32]. Some clinical studies suggest using clofazimine to replace rifampicin to compose the standard three-drug regimen [35,36], but the limited sample size and similar treatment efficacy with the current standard regimen require further studies. For fluoroquinolones, evidence linking MIC values to treatment outcomes is generally limited. Their activity against MAC varies between individual drugs and is further influenced by co-administered agents [37], which complicates the interpretation of MIC values in MAC-PD treatment. Collectively, although these agents have demonstrated activity against MAC [28], they may not be potent enough on their own to determine overall treatment success [38]. Their clinical efficacy should therefore be interpreted in the context of combination therapy, pharmacokinetic/pharmacodynamic (PK/PD) properties, tissue penetration, and drug–drug interactions.

Our study has the following limitations. First, the sample size valid for analysis is limited due to the low utilization of MIC testing in the study hospital over the study period, as well as the small number of cases within the high MIC categories. Therefore, our results should be interpreted as exploratory, and the observed associations between MICs and treatment outcomes may be influenced by potential confounders. Second, MICs were determined on a single occasion due to the cost. Inherent variations have been reported for the microdilution method used in the study, although not systematically biased [12,39]. Meanwhile, quality control was rigorously implemented in the study hospital to ensure the reliability of results. Third, the definition of clinical cure was based on radiologic improvement following the national guidelines [6], which may involve some subjectivity and may not always be consistent with the microbiological results. Fourth, *M. avium* was reported to have one-dilution-step higher ECOFFs than *M. intracellulare* for macrolides [12]. We did not perform additional subgroup analysis considering the limited sample size and the reality that MAC-PD patients were under an identical treatment principle regardless of specific subspecies. Fifth, the efficacy of a drug is not only decided by the susceptibility of a pathogen, but also other factors such as drug exposure. Patients with suboptimal drug exposure are at risk of acquisition of drug resistance and treatment failure despite their infected isolates being susceptible to treatment drugs. Therefore, PK/PD indices are believed to better explain the efficacy of a drug, and efforts should be made to establish PK/PD breakpoints for favorable outcome in future studies.

## 5. Conclusions

Our findings suggest an association between clarithromycin MIC above 64 mg/L and treatment outcomes in patients with MAC-PD receiving standard guideline-recommended regimens. Meanwhile, elevated ethambutol MICs also appeared to have potential clinical relevance. However, causal inference cannot be established, and the statistical power of our analysis was limited. Nevertheless, extremely high clarithromycin MICs and elevated ethambutol MICs may help identify patients at high risk of treatment failure with standard regimens. These patients may benefit from early treatment escalation and closer monitoring. In contrast, outcomes remained relatively consistent across different MIC levels for other antimycobacterial agents. The development of more potent agents and optimized regimens remains an urgent priority to further improve outcomes in patients with MAC-PD.

## Figures and Tables

**Table 1:** Definition for treatment outcomes of *Mycobacterium avium complex* pulmonary disease in this study.

Treatment Outcome	Definition from the NTM-NET Consensus Statement	Definition Used in This Study
Culture conversion	The finding of at least three consecutive negative mycobacterial cultures from respiratory samples, collected at least 4 weeks apart, during antimycobacterial treatment.	The finding of at least two consecutive negative mycobacterial cultures from sputum, collected at least 4 weeks apart, **or one negative mycobacterial culture from bronchial wash or lavage according to the Chinese national guidelines** [6], during antimycobacterial treatment.
Microbiological cure	Finding multiple consecutive negative but no positive cultures with the causative species from respiratory samples after culture conversion and until the end of antimycobacterial treatment.	No positive cultures with the causative species from respiratory samples after culture conversion and until the end of antimycobacterial treatment.
Clinical cure	Patient-reported and/or objective improvement of symptoms during antimycobacterial treatment, sustained until at least the end of treatment, but no cultures available to prove culture conversion or microbiological cure.	**Computerized tomography presents closure of pulmonary cavitation and resolution of pulmonary lesions**, sustained until at least the end of treatment, but no or not enough cultures available to prove culture conversion or microbiological cure.
Treatment failure	The re-emergence of multiple positive cultures or persistence of positive cultures with the causative species from respiratory samples after ≥12 months of antimycobacterial treatment while the patient is still on treatment.	The re-emergence of multiple positive cultures or persistence of positive cultures with the causative species from respiratory samples **or deteriorated pulmonary lesions on radiology when microbiological evidence is unavailable** after ≥12 months of antimycobacterial treatment while the patient is still on treatment.

NTM-NET: Nontuberculous Mycobacteria Network European Trials group. The sentences in bold are the main differences to the NTM-NET consensus statement.

**Table 2:** Demographic and clinical characteristics of study participants with *Mycobacterium avium complex* pulmonary disease.

	In Total (n = 209)
Age, years ^a^	60.0 (50.0, 66.0)
Male	63 (30.1)
Body mass index (n = 71) ^a^	18.9 (16.9, 20.9)
Subspecies	
* M. avium*	34 (16.3)
* M. intracellulare*	167 (79.9)
Both	8 (3.8)
Comorbidities	74 (35.4)
Diabetes type 2	12 (5.7)
Hypertension	21 (10.0)
Rheumatism	9 (4.3)
Chronic pulmonary diseases ^b^	19 (9.1)
Pulmonary fungus infection	20 (9.6)
Cancer	19 (9.1)
Erythrocyte sedimentation rate, mm/hr ^a^	35.0 (15.0, 65.0)
Bronchiectasis (n = 182)	161 (88.5)
Pulmonary cavitations (n = 182)	
0	110 (60.4)
1	26 (14.3)
≥2	46 (25.3)
Infected pulmonary zones (n = 182) ^a^	4.0 (3.0, 6.0)
Radiographic presentation (n = 182)	
Fibrocavitary	19 (10.4)
Noncavitary nodular bronchiectatic	98 (53.8)
Cavitary nodular bronchiectatic	46 (25.3)
Unclassifiable	19 (10.4)

Data are presented as number (percentage) unless otherwise specified. CT: computerized tomography. ^a^: presented as median (interquartile range). ^b^: sum of chronic obstructive pulmonary disease, idiopathic interstitial pneumonia, and pneumoconiosis.

**Table 3:** Treatment regimens and outcomes for patients initiating *Mycobacterium avium complex* pulmonary disease treatment.

	In Total (n = 209)
**Prior NTM treatment history**	27 (12.9)
Different NTM species	1 (0.5)
Identical NTM species	26 (12.4)
**Number of antimycobacterial drugs ^a^**	4.0 (3.0, 4.0)
**Adjustment of drug regimen**	67 (32.1)
**Reason for regimen adjustment**	
Adverse drug reactions	44 (21.1)
Poor treatment response	21 (10.0)
Drug resistance	9 (4.3)
Others	18 (8.6)
**Treatment duration, months ^a^**	15.7 (11.9, 19.3)
**Treatment outcome**	
Treatment success	104 (49.8)
Cure	51 (24.4)
Microbiological cure	12 (5.8)
Clinical cure	41 (19.6)
Treatment failure	51 (24.4)
Lost to follow-up	23 (11.0)
Treatment halted	21 (10.0)
Unevaluable	10 (4.8)
**Composite treatment outcome (n = 199)**	
Treatment success	104 (52.3)
Unfavorable outcome	95 (47.7)

Data are presented as number (percentage) unless otherwise specified. NTM: nontuberculous mycobacteria. ^a^: presented as median (interquartile range).

**Table 4:** Subgroup analysis on association between minimum inhibitory concentration (MIC) and treatment outcome in patients with *Mycobacterium avium complex* pulmonary disease (n = 97) ^a^.

	Treatment Outcome		OR (95% CI)
	Success Rate	Failure	
**Thresholds indicated in this study ^b^**			
Clarithromycin MIC, mg/L			
<64	76 (81.7)	17 (18.3)	1
≥64	1 (25.0)	3 (75.0)	13.4 (1.61–280.4)
Ethambutol MIC, mg/L			
≤16	74 (82.2)	16 (17.8)	1
>16	3 (42.9)	4 (57.1)	6.17 (1.25–34.0)
**Breakpoints suggested by the CLSI guidelines**			
Clarithromycin MIC, mg/L			
≤8	73 (81.1)	17 (18.9)	1
>8	4 (57.1)	3 (42.9)	3.22 (0.59–16.0)
Amikacin MIC, mg/L			
≤16	25 (78.1)	7 (21.9)	1
>16	6 (75.0)	2 (25.0)	1.19 (0.15–6.64)
Moxifloxacin MIC, mg/L			
≤1	4 (80.0)	1 (20.0)	1
>1	40 (81.6)	9 (18.4)	0.90 (0.11–18.7)

CLSI: Clinical and Laboratory Standards Institute; OR: odds ratio; CI: confidence interval. ^a^: the subgroup analysis was limited to patients who received the standard regimen (a macrolide, rifamycin, and ethambutol), with or without fluoroquinolone and/or amikacin, and completed the full treatment course. ^b^: The MIC thresholds for clarithromycin and ethambutol were indicated by the distribution of treatment success rate across different MIC levels in this study.

## Data Availability

The datasets used and analyzed during the current study are available from the corresponding author on reasonable request.

## Supplementary Materials

The following supporting information can be downloaded at https://www.mdpi.com/article/10.3390/pathogens14121218/s1. Table S1: Comparison of baseline demographic and clinical characteristics between patients with *Mycobacterium avium complex* pulmonary disease according to treatment outcomes; Table S2: Risk factors associated with the treatment outcome of patients with *Mycobacterium avium complex* pulmonary disease; Figure S1: Distribution of minimum inhibitory concentrations for 13 antimycobacterial drugs in clinical isolates of *Mycobacterium avium complex* at baseline (n = 209).
