# Supplementary material for: Association of Antimicrobial Susceptibility with Treatment Response in Mycobacterium avium complex Pulmonary Disease
Source: Pathogens. 2025 Nov 29;14(12):1218. doi: 10.3390/pathogens14121218 (PMC12736129; doi:10.3390/pathogens14121218)
Supplement: Supplementary file 1 [file pathogens-14-01218-s001.zip › pathogens-3968030-supplementary.pdf]

Table S1 Comparison of baseline demographic and clinical characteristics between patients with *Mycobacterium avium complex* pulmonary disease according to treatment outcomes

	Success/failure (n=155)	Lost to follow-up/treatment halted/unevaluable (n=54)	*P* value
Age, years [a]	59.0 (50.0, 64.5)	64.0 (49.8, 71.0)	**0.017**
Male	48 (31.0)	15 (27.8)	0.789
Body mass index [a]	18.8 (16.9, 20.6)	19.9 (18.4, 21.5)	0.413
Subspecies			
M. avium	25 (16.1)	9 (16.7)	0.281
M. intracellulare	122 (78.7)	45 (83.3)	
Both	8 (5.2)	0 (0.0)	
Comorbidities	55 (35.5)	19 (35.2)	1.000
Diabetes type 2	10 (6.5)	2 (3.7)	0.735
Hypertension	17 (11.0)	4 (7.4)	0.627
Rheumatism	5 (3.2)	4 (7.4)	0.241
Chronic pulmonary diseases [b]	15 (9.7)	4 (7.4)	0.786
Pulmonary fungus infection	14 (9.0)	6 (11.1)	0.858
Tumor	15 (9.7)	4 (7.4)	0.786
Erythrocyte sedimentation rate, mm/hr [a]	30.0 (15.0, 59.0)	52.5 (20.5, 85.8)	**0.038**
Prior NTM treatment history	15 (9.7)	12 (22.2)	**0.033**
Bronchiectasis	125 (88.7)	36 (87.8)	1.000
Pulmonary cavitation			
0	91 (64.5)	19 (46.3)	0.091
1	17 (12.1)	9 (22.0)	
≥2	33 (23.4)	13 (31.7)	
Infected pulmonary zones [a]	4.0 (3.0, 6.0)	4.0 (3.0, 6.0)	0.549
Radiographic presentation			
Fibrocavitary	14 (9.9)	5 (12.2)	0.092
Noncavitary nodular bronchiectatic	82 (58.2)	16 (39.0)	
Cavitary nodular bronchiectatic	30 (21.3)	16 (39.0)	
Unclassifiable	15 (10.6)	4 (9.8)	
Number of antimycobacterial drugs [a]	4.0 (3.0, 4.0)	4.0 (3.0, 4.0)	0.327
Adjustment of drug regimen	48 (31.0)	19 (35.2)	0.687

Data are presented as number (percentage) unless otherwise specified. NTM: non-tuberculous mycobacteria. [a]: presented as median (interquartile range). [b]: the sum of chronic obstructive pulmonary disease, idiopathic interstitial pneumonia and pneumoconiosis.

Table S2 Risk factors associated with the treatment outcome of patients with *Mycobacterium avium complex* pulmonary disease

	Treatment outcome			Composite treatment outcome		
	Success (n=104)	Failure (n=51)	*P* value	Success (n=104)	Unfavorable (n=95)	*P* value
Age, years [a]	56.5 (49.0, 64.0)	63.0 (54.5, 67.0)	**0.006**	56.5 (49.0, 64.0)	63.0 (52.5, 70.0)	**0.001**
Male	25 (24.0)	23 (45.1)	**0.013**	25 (24.0)	34 (35.8)	0.097
Body mass index [a]	19.3 (17.2, 21.6)	17.5 (16.6, 20.0)	**0.034**	19.3 (17.2, 21.6)	18.2 (16.7, 20.2)	0.100
Species						
M. avium	20 (19.2)	5 (9.8)	0.151	20 (19.2)	13 (13.7)	0.053
M. intracellulare	77 (74.0)	45 (88.2)		77 (74.0)	81 (85.3)	
Both	7 (6.7)	1 (2.0)		7 (6.7)	1 (1.1)	
Comorbidities	27 (26.0)	28 (54.9)	**0.001**	27 (26.0)	41 (43.2)	**0.016**
Diabetes type 2	6 (5.8)	4 (7.8)	0.730	6 (5.8)	5 (5.3)	1.000
Hypertension	9 (8.7)	8 (15.7)	0.297	9 (8.7)	11 (11.6)	0.653
Rheumatism	2 (1.9)	3 (5.9)	0.332	2 (1.9)	6 (6.3)	0.155
Chronic pulmonary diseases [b]	4 (3.8)	11 (21.6)	**0.001**	4 (3.8)	14 (14.7)	**0.015**
Pulmonary fungal infection	6 (5.8)	8 (15.7)	0.070	6 (5.8)	12 (12.6)	0.150
Cancer	12 (11.5)	3 (5.9)	0.388	12 (11.5)	6 (6.3)	0.300
Erythrocyte sedimentation rate, mm/hr [a]	25.0 (12.5, 49.0)	56.0 (20.5, 84.5)	**0.001**	25.0 (12.5, 49.0)	51.0 (20.0, 86.0)	**<0.001**
Prior NTM treatment history	9 (8.7)	6 (11.8)	0.570	9 (8.7)	14 (14.7)	0.263
Bronchiectasis	85 (88.5)	40 (88.9)	1.000	85 (88.5)	72 (88.9)	1.000
Pulmonary cavitation						
0	73 (76.0)	18 (40.0)	**<0.001**	73 (76.0)	34 (42.0)	**<0.001**
1	10 (10.4)	7 (15.6)		10 (10.4)	16 (19.8)	
≥2	13 (13.5)	20 (44.4)		13 (13.5)	31 (38.3)	
Infected pulmonary zones [a]	4.0 (2.0, 5.0)	6.0 (3.0, 6.0)	**<0.001**	4.0 (2.0, 5.0)	5.0 (3.0, 6.0)	**0.001**

Radiographic presentation						
Fibrocavitary	7 (7.3)	7 (15.6)	**<0.001**	7 (7.3)	11 (13.6)	**<0.001**
Noncavitary nodular bronchiectatic	64 (66.7)	18 (40.0)		64 (66.7)	32 (39.5)	
Cavitary nodular bronchiectatic	12 (12.5)	18 (40.0)		12 (12.5)	33 (40.7)	
Unclassifiable	13 (13.5)	2 (4.4)		13 (13.5)	5 (6.2)	
Number of antimycobacterial drugs [a]	4.0 (3.8, 5.0)	4.0 (3.0, 4.0)	**0.007**	4.0 (3.8, 5.0)	4.0 (3.0, 4.0)	**0.005**
Adjustment of drug regimen	31 (29.8)	17 (33.3)	0.794	31 (29.8)	32 (33.7)	0.664

Data are presented as number (percentage) unless otherwise specified. Patients with unevaluable outcome are excluded from analysis. Unfavorable outcome consists of treatment failure, lost to follow-up or treatment halted before treatment completion. NTM: non-tuberculous mycobacteria. [a]: presented as median (interquartile range). [b]: the sum of chronic obstructive pulmonary disease, idiopathic interstitial pneumonia and pneumoconiosis.

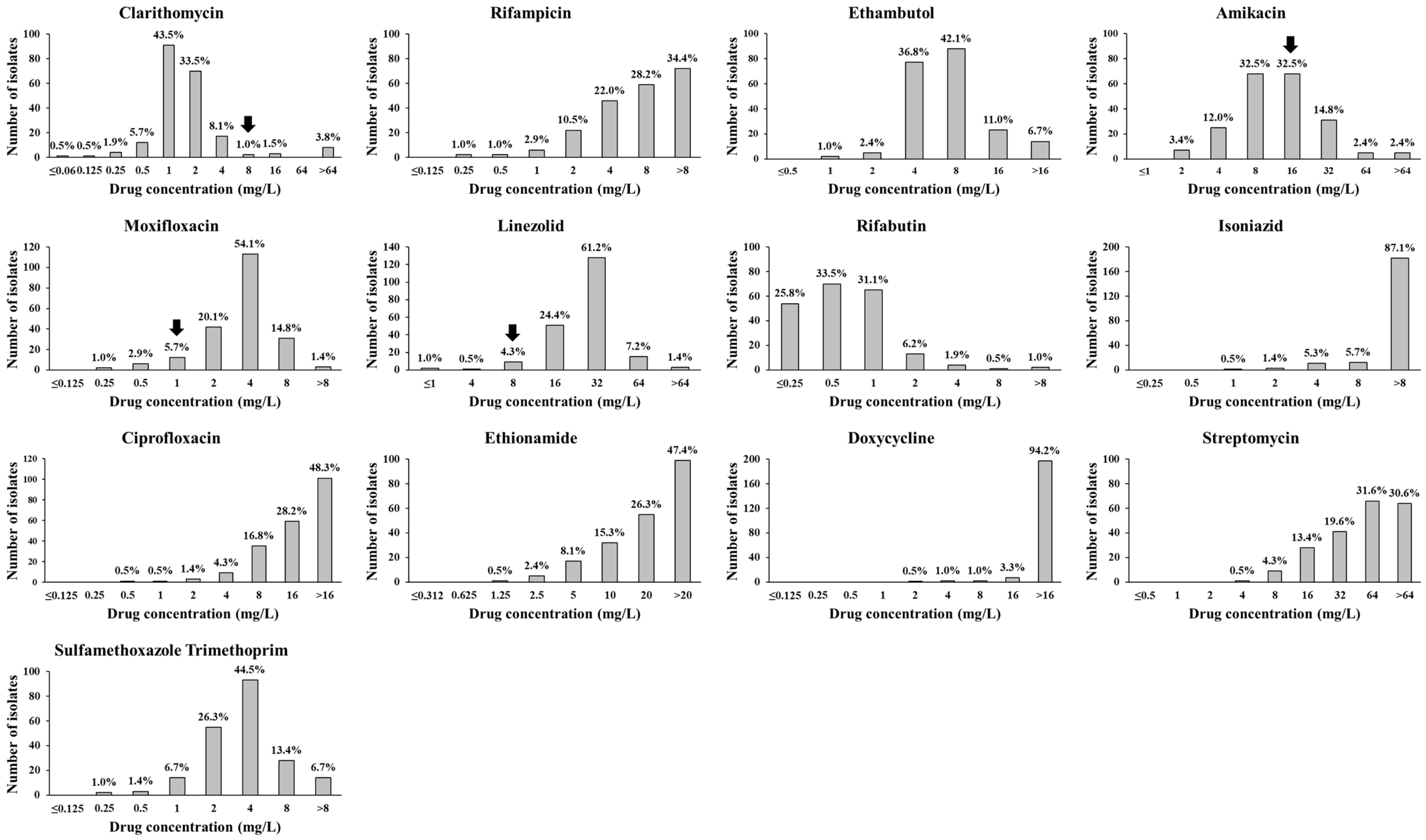

Figure S1. Distribution of minimum inhibitory concentrations for 13 antimycobacterial drugs in clinical isolates of *Mycobacterium avium* complex at baseline (n=209). The black arrows indicate the breakpoints suggested by the Clinical and Laboratory Standards Institute.
